# Oral Liquid Formulation of Levothyroxine Is Stable in Breakfast Beverages and May Improve Thyroid Patient Compliance

**DOI:** 10.3390/pharmaceutics5040621

**Published:** 2013-12-13

**Authors:** Alberto Bernareggi, Elia Grata, Maria Teresa Pinorini, Ario Conti

**Affiliations:** 1 Institut Biochimique SA (IBSA), Centro Insema, Via Cantonale, Manno CH-6928, Switzerland; 2 Alpine Foundation for Life Sciences (AFLS), Alpine Institute for Chemistry and Toxicology, Olivone CH-6718, Switzerland; E-Mails: elia.alpisti@bluewin.ch (E.G.); mtpin@ticino.com (M.T.P.); ario.conti@ti.ch (A.C.)

**Keywords:** levothyroxine, T4, liquid formulation, LC-MS/MS, stability, coffee, milk, tea, orange juice, bioavailability

## Abstract

Patients on treatment with levothyroxine (T4) are informed to take this drug in the morning, at least 30 min before having breakfast. A significant decrease of T4 absorption was reported, in fact, when T4 solid formulations are taken with food or coffee. According to preliminary clinical study reports, administration of T4 oral solution appears to be less sensitive to the effect of breakfast beverages on oral bioavailability. In the present study, stability of T4 oral solution added to breakfast beverages was investigated. A 1 mL ampoule of single-dose Tirosint^®^ oral solution (IBSA Farmaceutici Italia, Lodi, Italy) was poured into defined volumes of milk, tea, coffee, and coffee with milk warmed at 50 °C, as well as in orange juice at room temperature. Samples were sequentially collected up to 20 min and analyzed by validated liquid chromatography-tandem mass spectrometry (LC-MS/MS) methods. The results of the study demonstrated that T4 is stable in all beverages after 20 min incubation. Demonstration of T4 stability is a prerequisite for a thorough evaluation of the effect of breakfast beverages on the bioavailability of T4 given as oral solution and for a better understanding of the reasons underlying a decreased T4 bioavailability administered as solid formulations.

## 1. Introduction

Levothyroxine (T4) is a thyroid hormone secreted by the follicular cells of the thyroid involved in a number of physiological functions, including growth, cardiac functionality, and regulation of carbohydrates, lipid, and protein metabolism. Levothyroxine sodium is the synthetic hormone commonly prescribed to treat some of thyroid diseases, particularly as a replacement therapy for thyroid hormone deficiency (hypothyroidism) of any etiology and for pituitary thyroid stimulating hormone (TSH) suppression [1].

T4 formulations on the market are represented by solid formulations (tablets, softgel capsules) and also multiple- and single-dose liquid formulations for oral use. Tirosint^®^ single-dose oral solution (IBSA Farmaceutici Italia, Lodi, Italy) consists of a 1 mL solution of T4 dissolved at concentrations of 25, 50, 75, and 100 µg/mL in a mixture of 85% glycerol and 96% ethanol. The solution is supplied in squeezable low-density polyethylene (LDPE) ampoules. The most important advantage of an oral solution, as compared to the solid formulation, is the ease of administration, which makes it particularly suitable for patients who are not able to swallow intact capsules or tablets.

Levothyroxine is a narrow therapeutic index drug that requires dose individualization. Patients have varying degrees of hypothyroidism and varying responses to the medication. As a result, levothyroxine dose must be individualized to warrant the desired efficacy and minimize the unwanted side effects. Optimal dosing is set on the basis of clinical response and biochemical tests, including monitoring of TSH serum levels. 

In order to provide patients with accurate doses, T4 formulations need to be administered under conditions that do not affect T4 bioavailability. Patients on treatment with T4 tablet formulations are usually informed to take the drug in the morning in fasting conditions, prior to breakfast, to avoid possible interactions with food [2,3,4,5,6], dietary fibers [7,8], coffee [9], coffee with milk [4], and other breakfast drinks [10] reported in the literature. 

Oral administration of T4 tablets in fasting conditions may ensure better efficacy (e.g., lowering of TSH serum levels) as compared to non-fasting administration modalities that may imply decrease in T4 bioavailability. Non-fasting regimens of levothyroxine oral administration of solid formulations are reported to be associated to significantly higher serum TSH concentrations. This finding is interpreted as due to a more favorable T4 absorption from the gastrointestinal tract in fasting conditions [4].

The interference on levothyroxine absorption observed after T4 tablet administration with coffee appears to be reduced by replacing tablets with T4 softgel capsules [11,12,13]. This may be explained considering that the softgel capsule consists in a gelatine-based shell-wall containing T4 dissolved in a hydrophylic viscous liquid. Interaction of T4 with coffee is therefore prevented by the presence of the capsule shell until the shell opens up in the stomach. After capsule shell disgregation, T4 becomes immediately available for absorption. A case of impaired absorption of T4 tablets induced by the proton pump inhibitor pantoprazole was solved by switching the patient to T4 softgel capsules. This finding may be explained on the same ground, *i.e.*, the bioavailability of T4 administered as softgel capsules is less affected, if any, by changes in the gastric pH as compared to the T4 tablet [14].

Prompt bioavailability of T4 is even more expected in case T4 is administered as an oral solution [13,15,16]. Indeed, in a few patients who switched from T4 tablet to T4 oral solution keeping the habit of having coffee concomitantly or within 5 min after T4 ingestion, a decrease in serum TSH was observed [13]. In patients who underwent bariatric surgery, a change from T4 tablets to T4 oral solution proved to normalize serum TSH levels [15]. Switching back to tablets caused TSH levels to worsen. It was also reported that in euthyroid patients who used to take a liquid T4 formulation with coffee at breakfast, TSH, free levothyroxine (fT4), and (free triiodothyronine) fT3 serum levels did not change significantly, after three and six months of treatment, if the T4 solution administration was switched to thirty minutes before breakfast [16].

We could speculate that the rapid absorption of T4 from oral liquid formulations may overcome or reduce the interaction with certain food and breakfast drinks. These findings open interesting perspectives that require further evaluations.

Recovery tests on known concentrations of T4 (20 or 33 ng/mL) were performed *in vitro* in the presence of saline, freshly brewed espresso, or known T4 sequestrants (dietary fibers from bran, aluminium hydroxide/magnesium hydroxide, and sucralfate) [9]. While in saline, T4 recovery was complete, T4 recovery after dilution of T4 solution in coffee ranged from 56% to 80%. Recovery of T4 from suspensions of dietary fibers, aluminium hydroxide/magnesium hydroxyde, and sucralfate ranged from 17% to 47%. Incomplete recovery may suggest either T4 sequestration or T4 degradation.

The purpose of the present study was to test levothyroxine stability by using a single-dose solution (Tirosint^®^ oral solution, 1 mL) after its dilution in different hot beverages, namely milk, tea, coffee, coffee with milk, and in orange juice at room temperature, conditions that simulate the administration of T4 with beverages during breakfast. After T4 addition, samples were sequentially collected up to 20 min and analyzed by validated liquid chromatography-tandem mass spectrometry (LC-MS/MS) methods. Demonstration of T4 stability is a prerequisite for a thorough *in vivo* evaluation of the potential interaction between breakfast beverages and T4 absorption when T4 is given as an aqueous solution.

## 2. Experimental Section

### 2.1. Chemicals and Reagents

Levothyroxine sodium was provided by IBSA (Institut Biochimique, Manno, Switzerland) while [^13^C_6_]L-thyroxine (tyrosine-ring-^13^C_6_, 99% purity) was purchased from Cambridge Isotope Laboratories, Inc. (Tewksbury, MA, USA) for use as internal standard (IS). HPLC-grade methanol and acetonitrile were acquired from Carlo Erba (Milano, Italy), while formic acid and potassium dihydrogen phosphate were purchased from Sigma-Aldrich (Buchs, Switzerland). Ultrapure water was prepared in house from deionized water with a MilliQ System (Millipore, Billerica, MA, USA).

### 2.2. Levothyroxine Oral Solution

Levothyroxine oral solution (Tirosint^®^ 100 µg/mL oral solution) was provided by IBSA Farmaceutici Italia (Lodi, Italy). The formulation consists in a mixture of 96% ethanol and 85% glycerol in which T4 is dissolved at the concentration of 100 µg/mL. The solution is supplied in squeezable low density polyethylene ampoules (primary packaging) filled to a nominal volume of 1.0 mL (+10% overfilling). A strip of five single-dose ampoules is packaged in a coupled PET/Alu/PE envelope (secondary packaging).

### 2.3. Matrices (Beverages)

Cow milk 1.5% fat (Drink UHT, ultra-high temperature processing), Lavazza red label coffee, Lipton Yellow Label Tea (Black Tea), orange juice (“Michel type”, 100% fruit) were purchased from local distributors (Denner, Olivone, Switzerland). Coffee was prepared with a 0.3 L coffee maker.

### 2.4. Instrumentation and Analytical Conditions

Analyses were performed by an Accela HPLC system equipped with a TSQ Vantage triple quadrupole mass spectrometer (ThermoFisher Scientific, Waltham, MA, USA) used in ESI positive polarity mode. An Accucore RP-MS, 50 × 2.1 mm, 2.6 µm, column (ThermoFisher Scientific, Waltham, MA, USA) with an Accucore RP-MS, 10 × 2.1 mm, 2.6 µm pre-column (ThermoFisher Scientific, Waltham, MA, USA) were used for chromatographic separations. The mobile phase consisted of water + 0.1% formic acid (solvent A) and acetonitrile + 0.1% formic acid (solvent B) delivered at a flow rate of 0.5 mL/min. The mobile phase gradient is summarized in Table 1.

**Table 1:** Mobile phase gradient used for chromatographic separation.

Time (min)	Eluent A (%)	Eluent B (%)
0.00	98.0	2.0
2.00	2.0	98.0
2.40	2.0	98.0
2.50	98.0	2.0
3.00	98.0	2.0

The analytical column was maintained at 35 °C, and extracted samples kept at 15 °C while inside the autosampler. The injection volume was 15 µL. The autosampler needle was washed with a mixture 50:50 methanol-water (*v*/*v*) solution between injections.

A TSQ Vantage triple quadrupole mass-spectrometer (ThermoFisher Scientific, Waltham, MA, USA) interfaced with a positive electrospray ionization source (HESI-II Probe, ThermoFisher Scientific, Waltham, MA, USA) was used for the detection. The ion source parameters were set as follows: ionspray voltage 4000 V, capillary temperature 280 °C, vaporizer temperature 325 °C, sheath gas pressure 45.0, aux valve flow 15.0, collision gas pressure 1.5 mTorr. All analytes and internal standard were detected in selected reaction monitoring (SRM) mode as shown in Table 2.

**Table 2:** Optimized parameters for selected reaction monitoring (SRM).

Parent	Product	CollisionEnergy	Start	Stop	s-lens	Name
651.900	197.112	52	0.29	2.25	107	Triiodothyronine
651.900	224.150	37	0.29	2.25	107	Triiodothyronine
651.900	606.016	9	0.29	2.25	107	Triiodothyronine
777.865	324.066	39	1.37	2.07	139	Levothyroxine (T4)
777.865	351.091	32	1.37	2.07	139	Levothyroxine
777.865	732.002	9	1.37	2.07	139	Levothyroxine
783.810	357.102	49	1.37	2.07	180	[^13^C_6_]L-thyroxine
783.810	640.045	25	1.37	2.07	180	[^13^C_6_]L-thyroxine
783.810	737.960	25	1.37	2.07	180	[^13^C_6_]L-thyroxine

Data acquisition, processing and storage were performed using Xcalibur software, version 2.1 (ThermoFisher Scientific, Waltham, MA, USA). Calculations were based on peak area ratios of analyte to internal standard.

### 2.5. Preparation of Calibration Standards, Internal Standards, and Quality Controls

Stock solution of T4 was prepared by dissolving sodium levothyroxine in methanol at the concentration of 1 mg/mL. A second stock solution of T4 was prepared by diluting the original T4 solution with methanol to a concentration of 1 µg/mL. A stock solution of the internal standard (IS) was prepared by dissolving 0.10 mg of [^13^C_6_]L-thyroxine in the original vial with 1 mL of methanol to the concentration of 100 µg/mL. A second IS stock solution was prepared by diluting the original IS solution with methanol to a concentration of 10 µg/mL. The stock IS solutions were stored at −20 °C prior to use. Calibration samples of levothyroxine were prepared in each matrix, namely, milk, coffee, tea, coffee with milk 4:1 *v*/*v* and orange juice, at six different concentrations: 0.4, 0.6, 0.8. 1.0 and 1.2 µg/mL. Quality control (QC) samples of T4 were also prepared in each matrix at low (0.4 µg/mL), medium (1.0 µg/mL), and high (1.4 µg/mL) concentrations.

### 2.6. Stability Samples Preparation

Milk, tea, coffee, coffee with milk, and orange juice were freshly prepared. The temperature of milk, tea, coffee, and coffee with milk was increased until the samples reached 50 °C. Orange juice was used at room temperature. The content (1 mL) of single-dose T4 solution was squeezed into 99 mL of each selected beverage. Each ampoule was then rinsed with the beverage to remove also the 10% overfilling. Therefore, the expected T4 concentration in each matrix was 1.1 µg/mL. After gentle mixing, sample aliquots of 100 µL were withdrawn in triplicate from each matrix after 0, 2, 5, 10, 15, and 20 min from preparation.

### 2.7. Sample Extraction

Validation and stability samples were prepared using solid phase extraction (SPE). Each sample (100 µL) was placed in a 1 mL-collection plate containing 10 µL of a 10 µg/mL IS solution and 300 µL of a 0.1 M dihydrogen phosphate buffer solution pH = 7. An Evolute ABN Express 30 mg plate (Biotage, Uppsala, Sweden) was placed on a Pressure + 96 positive pressure manifold (Biotage, Uppsala, Sweden) with waste plate in position. Aqueous samples were transferred to each well and a pressure of 2 psi was applied. Once samples were loaded, a washing step with 500 µL of a 0.1 M dihydrogen phosphate buffer solution pH = 7 was applied. Elution was performed by adding 2 mL of methanol with the collection plate in position and applying a pressure of 2 psi. Finally, a vacuum of 5 psi was maintened for 2 min and the organic phase was evaporated under nitrogen. Residues were reconstituted in 100 µL of methanol/water (95:5, *v*/*v*).

### 2.8. Method Validation

LC-MS/MS methods developed for the assay of T4 in each individual matrix were validated by determining the following parameters: selectivity, linearity, intra-assay precision and accuracy, recovery, and matrix effects. The validation approach used is justified for the intended purpose of the investigation where the methods were applied, *i.e.*, the intra-day stability of levothyroxine in beverages.

### 2.9. Abbreviations

The following abbreviations are used in the text: 

*C*_cal_ (µg/mL) = calculated concentration;

*C*_mean_ (µg/mL) = mean of calculated concentrations = ∑*C*_cal_/*N*;

*C*_exp_ (µg/mL) = expected (spiked) concentration; 

*C*_exp,_ _mean_ (µg/mL) = mean of expected (spiked) concentrations;

*Bias* (%) = 100 × (*C*_cal_ − *C*_exp_)/*C*_exp_;

*Bias*_mean_ (%) = 100 × (*C*_mean_ − *C*_exp_)/*C*_exp_;

*LLOQ* (µg/mL) = lower limit of quantification;

*ULOQ* (µg/mL) = upper limit of quantification;

*RE* (%) = accuracy, expressed as relative error percent;

*CV* (%) = precision, expressed as intra-assay coefficient of variation = 100 × *SD*/*C*_mean_.

## 3. Results and Discussion

### 3.1. Method Validation

#### 3.1.1. Specificity

**Figure 1:**
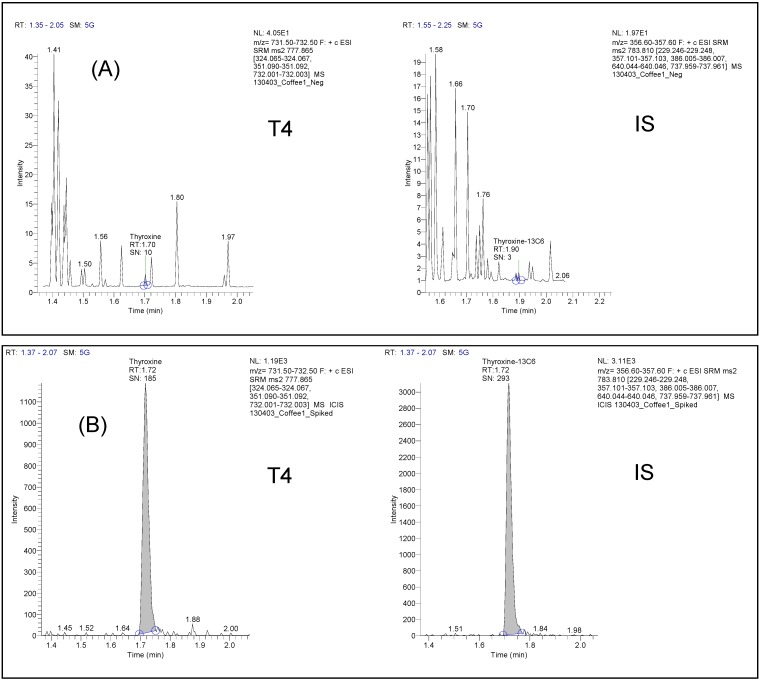
Representative LC-MS/MS chromatograms of T4 and IS obtained for (**A**) a coffee blank matrix and (**B**) a coffee QC spiked with low T4 and IS concentrations (0.4 µg/mL).

It was assessed for both T4 and the IS from three different blank matrix sources of each selected beverage. Possible interferences were evaluated by comparing the mean response of the blank samples against the mean response of the extracted QC *LLOQ* samples at the retention time of the analyte. Specificity was proven for milk, coffee, tea, coffee with milk, and orange juice. No interfering peaks appeared at the retention times of the analyte and the IS in these samples (Figure 1). In particular, the mean areas of peaks interfering with T4 determination accounted for 1.4% (milk), 0.4% (tea), 0.3% (coffee), 0% (coffee + milk), and 0.1% (orange juice) of mean T4 areas at *LLOQ*.

#### 3.1.2. Linearity and Quantification Limit

**Figure 2:**
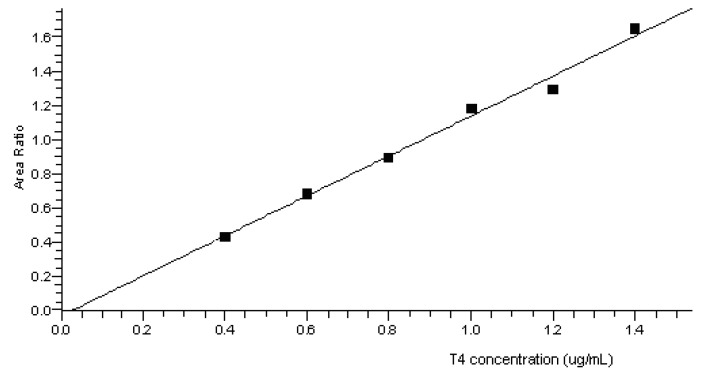
Representative calibration curve for T4 in coffee.

Increasing amounts of working solutions of the analyte were added to each blank matrix. Linearity was evaluated by analyzing three replicates of spiked blank samples at 0.4 (*LLOQ*), 0.6, 0.8, 1.0, 1.2, and 1.4 µg/mL. For each matrix, the six-point calibration curves were linear over the whole range. Determination coefficients *R*^2^ ranged between 0.98 and 0.99. In all tested matrices, *bias* and *bias*_mean_ values for at least 75% of the back calculated concentrations were within ±15.0% of the expected concentrations. Linearity parameters are reported in Table 3 and an example of a calibration curve set up for T4 in coffee is presented in Figure 2.

Based on a sample volume of 100 µL, the quantification range started from a *LLOQ* of 0.4 µg/mL leading up to an *ULOQ* of 1.4 µg/mL.

**Table 3:** Linearity parameters determined for each matrix.

Parameter	Result
*Bias*_mean_ at the *LLOQ* (0.4 µg/mL, *N* = 3 per matrix)	Milk: 2.2%
	Tea: 5.3%
	Coffee: 2.3%
	Coffee with milk: −1.0%
	Orange juice: 1.4%
*Bias*_mean_ above the *LLOQ* (0.6, 0.8, 1.0, 1.2 and 1.4 µg/mL, *N* = 3 per matrix)	Milk: from −3.3% to 1.3%
	Tea: from −2.5% to 1.1%
	Coffee: from −6.1% to 4.2%
	Coffee with milk: from −2.4% to 2.7%
	Orange juice: from −0.22% to 4.3%
Precision (*CV*%) at the *LLOQ* (0.4 µg/mL, *N* = 3 per matrix)	Milk: 3.3%
	Tea: 10%
	Coffee: 9.2%
	Coffee with Milk: 12%
	Orange juice: 1.9%
Precision (*CV*%) above the *LLOQ* (0.6, 0.8, 1.0, 1.2 and 1.4 µg/mL, *N* = 3 per matrix)	Milk: from 0.0% to 4.7%
	Tea: from 2.6% to 11.2%
	Coffee: from 1.6% to 7.9%
	Coffee with milk: from 0.69% to 6.8%
	Orange juice: from 0.66% to 8.4%

#### 3.1.3. Accuracy and Precision

Intra-assay accuracy and precision of the method were assessed by analyzing six replicates of QC samples at three concentration levels (0.4, 1.0 and 1.4 µg/mL) on a single day for each matrix. The results are reported in Table 4A,B. According to the Food and Drug Administration (FDA) Guidance for Industry on bioanalytical method validation [17], the results were within the acceptance criteria. Indeed, the intra-assay accuracy varied between 95.7% and 104% (accepted range: 85%–115%) and the precision between 3.0% and 7.8% (accepted limit, ±20% at *LLOQ* and ±15% above the *LLOQ*).

#### 3.1.4. Recovery and Matrix Effects

The extraction recovery and matrix effects were determined according to Matuszewski *et al.* [18]. Extraction recovery was evaluated by comparing the analyte/internal standard areas ratio of pre-extraction spiked samples to those of post-extraction spiked samples. 

Matrix effects were determined by comparing the analyte response of post-extraction spiked samples to those of pure standards representing 100% recovery. Three replicates at low (0.4 µg/mL), medium (1.0 µg/mL), and high (1.4 µg/mL) concentration levels were analyzed. Recovery values of T4 from the different matrices are reported in Table 5.

**Table 4:** (**A**) Intra-assay precision and accuracy for the determination of levothyroxine (T4) in milk, tea and coffee; (**B**) Intra-assay precision and accuracy for the determination of T4 in coffee with milk and orange juice.

Matrix	Parameter	QC Low (0.4 µg/mL)	QC Medium (1.0 µg/mL)	QC High (1.4 µg/mL)
**A**				
**Milk**	*C*_cal_ (µg/mL)	0.434	0.983	1.396
		0.401	0.996	1.403
		0.422	1.063	1.351
		0.392	1.012	1.465
		0.418	0.992	1.392
		0.414	1.062	1.458
	*C*_exp_ (µg/mL)	0.40	1.0	1.4
	*C*_mean_ (µg/mL)	0.41	1.0	1.4
	*RE* (%)	103	102	101
	*CV* (%)	3.6	3.5	3.0
**Tea**	*C*_cal_ (µg/mL)	0.456	0.893	1.333
		0.453	0.971	1.406
		0.397	1.049	1.494
		0.422	0.944	1.382
		0.373	0.942	1.444
		0.405	1.017	1.526
	*C*_exp_ (µg/mL)	0.40	1.0	1.4
	*C*_mean_ (µg/mL)	0.42	1.0	1.4
	*RE* (%)	104	96.9	102
	*CV* (%)	7.8	5.8	5.0
**Coffee**	*C*_cal_ (µg/mL)	0.395	0.995	1.353
		0.458	0.936	1.452
		0.390	1.142	1.502
		0.392	1.034	1.515
		0.392	1.060	1.321
		0.458	0.972	1.389
	*C*_exp_ (µg/mL)	0.40	1.0	1.4
	*C*_mean_ (µg/mL)	0.41	1.0	1.4
	*RE* (%)	104	102	102
	*CV* (%)	8.2	7.2	5.6
**B**				
**Coffee with Milk**	*C*_cal_ (µg/mL)	0.370	1.084	1.329
		0.399	0.997	1.364
		0.398	0.909	1.437
		0.374	0.916	1.486
		0.392	1.027	1.358
		0.410	0.956	1.236
	*C*_exp_ (µg/mL)	0.40	1.0	1.4
	*C*_mean_ (µg/mL)	0.39	1.0	1.4
	*RE* (%)	97.6	98.2	97.8
	*CV* (%)	4.0	6.9	6.3
**Orange juice**	*C*_cal_ (µg/mL)	0.389	0.962	1.315
		0.385	0.982	1.468
		0.451	0.973	1.366
		0.349	1.071	1.217
		0.369	0.986	1.346
		0.358	0.943	1.327
	*C*_exp_ (µg/mL)	0.40	1.0	1.4
	*C*_mean_ (µg/mL)	0.38	1.0	1.3
	*RE* (%)	95.9	98.6	95.7
	*CV* (%)	9.5	4.5	6.1

**Table 5:** Recovery of T4 from the different beverages.

Matrix	QC Low 0.4 µg/mL (%)	QC Medium 1.0 µg/mL (%)	QC High 1.4 µg/mL (%)
Milk	70	73	84
Tea	90	80	83
Coffee	79	81	102
Coffee + Milk	71	92	91
Orange juice	82	82	82

The calculated matrix effects varied from 1.1 to 1.2 for milk, from 1.0 to 1.3 for tea, from 0.18 to 0.34 for coffee; from 0.52 to 0.62 for coffee + milk and from 1.7 to 1.8 for orange juice. Ion suppression was observed for coffee and coffee with milk, ion enhancement for orange juice. No significant effect on MS response was highlighted for milk and tea. 

### 3.2. Stability of T4 in Beverages

The aim of this study was the stability evaluation of T4 formulated as a single-dose oral solution after its dilution in different beverages, namely milk, tea, coffee, coffee with milk and orange juice. 

**Figure 3:**
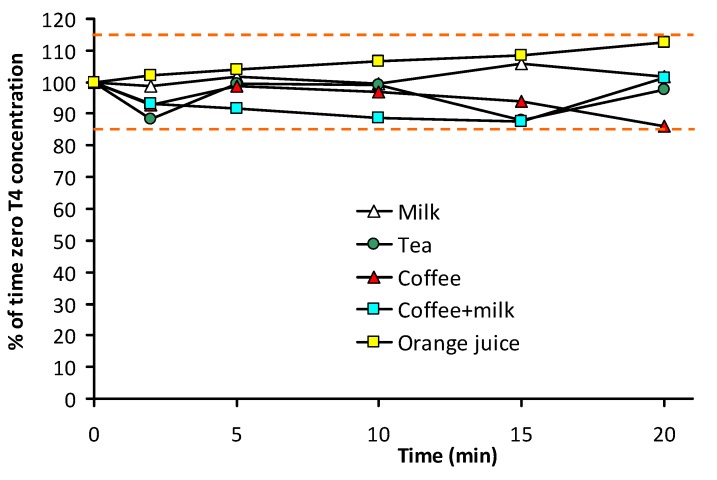
Graphical representation of mean percent variation of T4 concentration as a function of time with respect to time zero concentration for all tested beverages. Dashed lines indicate the acceptance limits of ±15% of time zero concentrations.

For each selected matrix, test were performed in triplicate. The mean T4 concentration values observed at each collection time were expressed as percentage of the initial mean concentration. The results are reported in Table 6 and Figure 3.

**Table 6:** Stability of levothyroxine in breakfast beverages. Mean assay values (and *CV*) are expressed as percentage of mean time zero concentrations.

Time (min)	Milk	Tea	Coffee	Coffee + Milk	Orange juice
0	100.0 (0.6)	100.0 (9.9)	100 (5.8)	100 (13.0)	100 (10.1)
2	98.8 (2.9)	88.5 (5.6)	92.6 (3.4)	93.1 (2.4)	102.0 (2.5)
5	101.7 (2.3)	99.4 (15.6)	98.7 (9.1)	91.6 (3.9)	104.0 (5.6)
10	99.5 (4.4)	99.1 (7.2)	96.9 (2.0)	88.7 (4.1)	106.5 (1.2)
15	106.0 (5.1)	87.9 (2.9)	93.8 (7.4)	87.6 (1.5)	108.5 (6.1)
20	101.7 (5.1)	97.8 (6.1)	85.9 (4.4)	101.2 (10.4)	112.7 (10.5)

## 4. Conclusions

In the present study, LC-MS/MS analytical methods have been developed and partially validated for the determination of T4 concentrations in breakfast beverages, such as milk, tea, coffee, coffee with milk, and orange juice. Methods involved solid phase extraction and electrospray positive ionization MS detection. Methods proved to be suitable for the quantification of T4 in those matrices and for application to the investigation of T4 stability. 

Stability of levothyroxine oral solution in milk, tea, coffee, and coffee with milk warmed to 50 °C, and in orange juice at room temperature was demonstrated up to 20 min after squeezing a 1 mL single-dose Tirosint^®^ ampoule in each of the matrices. With respect to the time zero concentrations, T4 assays ranged from 98.8% to 106% in milk, from 87.9% to 99.4% in tea, from 85.9% to 98.7% in coffee, from 87.6% to 101.2% in coffee + milk, and from 102% to 112.7% in orange juice. Considering an accepted analytical variation of ±15%, we can conclude that no evidence of T4 degradation occurred in all matrices. Moreover, in all matrices no trend to T4 concentration decrease as a function of the incubation time was observed. 

In conclusion, this study demonstrated that levothyroxine is stable in various hot beverages and in orange juice for at least 20 min. 

Demonstration of T4 stability in breakfast beverages represents a prerequisite for further investigations aimed at determining whether concomitant intake of T4 oral solution with breakfast beverages may affect T4 *in vivo* bioavailability, as already ascertained by different authors for tablet T4 formulations.
